# The interplay between epidermal cells and the cutaneous sensory nervous: a systematic review

**DOI:** 10.3389/fimmu.2026.1809838

**Published:** 2026-05-26

**Authors:** Feifei Wang, Xiaohong An, Dongjie Fan, Jing Shang

**Affiliations:** 1 School of Traditional Chinese Pharmacy, China Pharmaceutical University, Nanjing Jiangsu, China; 2 Yunnan Characteristic Plant Extraction Laboratory, Yunnan Characteristic Plant Extraction Laboratory Co., Ltd., Kunming, Yunnan, China; 3 Botanee Research Institute, Yunnan Botanee Bio-Technology Group Co., Ltd, Kunming, Yunnan, China; 4 Botanee Research Institute, Shanghai Jiyan Bio-pharmaceutical Co., Ltd., Shanghai, China

**Keywords:** cutaneous sensory nervous, epidermal cells, interaction, langerhans cell, mechanical allodynia, mechanical itch

## Abstract

The skin is the largest sensory organ in the human body. It has a neural structure that’s very complex and diverse. A neural network spreads through the epidermis, dermis, and subcutaneous tissues. This network generates an exceedingly complex structure. It shows the deep functional links between the cutaneous system and the nervous system. This review systematically delineates the distribution patterns of cutaneous sensory nerves, elaborates on their distinct functional properties, and explores in depth the bidirectional regulatory crosstalk between epidermal cells and the sensory nervous system. Direct communication between these cells and nerves is not the only focus. The review expands on the potential mechanisms underlying these interactions, including chemical signal transmission and the regulation of immune responses. The review further addresses the role of sensory nerves in dermatological pathology, encompassing their functions in processes like chronic inflammation, pain perception, wound healing, pigmentation, and other related ones.

## Introduction

The skin contains complex neural networks. It performs a variety of physiological activities, including barrier protection, sensory perception, immune defense, thermoregulation, endocrine activity, and excretion. Additionally, it shields the body from mechanical harm. It also fends off pathogenic infections, prevents dehydration, and copes with body temperature changes (1, 2). Think about the nice feeling of a soft breeze on your face. Or the sharp pain when you get close to something hot. All these experiences happen because of sensory signals transmitted through cutaneous nerves.

The skin contains a dense network of nerve endings composed of terminals from a variety of sensory neurons. For example, nociceptors pick up harmful stimuli and send pain signals. Thermoreceptors detect temperature changes. Low-threshold mechanoreceptors (LTMRs) sense light touches or non-painful physical pressure (3). The epidermis is the skin’s outermost layer. It not only acts as a barrier but also plays an active role in regulating nerve activity. Most epidermal cells express receptors for neuropeptides, which enable them to receive signaling molecules secreted by sensory neurons. Similarly, epidermal cells are capable of producing a variety of bioactive substances, including cytokines, growth factors, and neurotransmitters, which in turn can modulate the functional activity of sensory nerves. This bidirectional crosstalk creates a dynamic feedback loop between epidermal cells and sensory neurons, allowing for coordinated regulation of the body’s reactions to external stimuli (4).

Both the skin and the nervous system originate from the ectoderm during embryonic development. This shared developmental lineage provides a structural basis for close communication between certain epidermal cells and neurons (5). Epidermal cells and sensory neurons are in close anatomical proximity and share conserved signaling pathways, enabling tight bidirectional crosstalk (6). Such interactions provide a reasonable biological basis for the clinical comorbidity between cutaneous disorders and neurological diseases. For example, neurological dysfunctions may exacerbate inflammatory dermatoses such as psoriasis and atopic dermatitis (AD). Conversely, chronic cutaneous pathologies are associated with increased anxiety, sleep disturbances, and elevated susceptibility to neurodegenerative diseases. Although skin disorders are unlikely to serve as primary etiology of neurological diseases, they may act as contributing factors that accelerate and aggravate neuropathological progression.

This review examines the distribution of neural networks in the skin, explores and delineates the intricate regulatory mechanisms governing the crosstalk between epidermal cells and sensory neurons interact. By understanding these complex pathways, we might study potential mechanisms of action, thereby providing insights into the prevention and treatment of neurogenic skin diseases.

## Skin structure

The skin is a multilayered organ that includes the epidermis, dermis, subcutaneous tissue, and appendages. As the outermost layer of the skin, the epidermis consists of stratified squamous epithelium, which undergoes continuous proliferation and self-renewal. Histologically, the epidermis can be divided into five distinct layers, including the stratum corneum, stratum lucidum, granular layer, spinous layer, and stratum basale (7). Keratinocytes are the most common cells in the epidermis. Undifferentiated keratinocytes are capable of rapid growth and renewal. They gradually change to form the suprabasal layers, which include the spinous layer, granular layer, and stratum corneum (8, 9). The epidermis also has other special cells, including melanocytes, langerhans cells (LCs), Merkel cells and free nerve endings (FNEs).

The epidermal rete ridges interdigitate with the dermal papillary projections, with both structures anchored to the basement membrane (10, 11). The dermis lies under the epidermis and consists of two distinct layers: the papillary and reticular dermis. The dermis serves as the supportive framework of the skin, providing it with mechanical strength, nutritional support, and immune regulatory functions (12). The papillary dermis is composed of papillary fibroblasts. It contains many capillaries, lymphatic channels, FNEs, and Meissner corpuscles (1, 11). Papillary fibroblasts produce important components of the extracellular matrix, including type VI collagen, fibronectin, and tenascin C (13–15). The reticular layer is composed of reticular fibroblasts. It consists of densely packed collagen and elastic fibers, larger blood vessels, lymphatic vessels, nerves, and skin appendages, which collectively endow the skin with mechanical strength and elastic resilience (16, 17). The dermis also contains additional immune cells, including lymphocytes, macrophages, mast cells, and dendritic cells (7).

The reticular fibroblast progenitors can produce both adipocyte precursors and mature adipocytes, which collectively contribute to the formation of cutaneous adipose tissue (18, 19). Sweat glands, hair follicles, blood arteries, lymphatic vessels, and nerves are all dispersed throughout the dermal adipose tissue. They serve as a cushion against external injury, regulate body temperature, store energy, and contribute to the body’s natural immune defense against bacterial pathogens (20).

Skin appendages—such as hair follicles, sweat glands, and sebaceous glands —are embedded within the dermis and extend into the subcutaneous tissue (21). The hair follicle is a complex sheath-like structure that surrounds the hair root and consists of multiple components, which spans from the subcutaneous tissue to the epidermis (1). It includes the hair shaft, root sheath, hair matrix, and hair papilla, in which the hair matrix consists of clusters of epidermal cells, while the hair papilla is a connective tissue projection extending into the hair matrix and richly supplied with blood vessels and nerves (21). Sebaceous glands are typically composed of one or more secretory acini connected to a short common duct. Most are situated adjacent to hair follicles and the arrector pili muscle, although some exist independently in non-follicular regions of the skin, such as the meibomian glands of the eyelid (22). Sweat glands are the primary effectors of thermoregulation and perspiration (23). They consist of two main parts: a secretory segment located in the deep dermis or subcutaneous tissue, and an excretory duct that traverses the epidermis to open directly onto the skin surface.

## Cutaneous nerves

**Figure 1:**
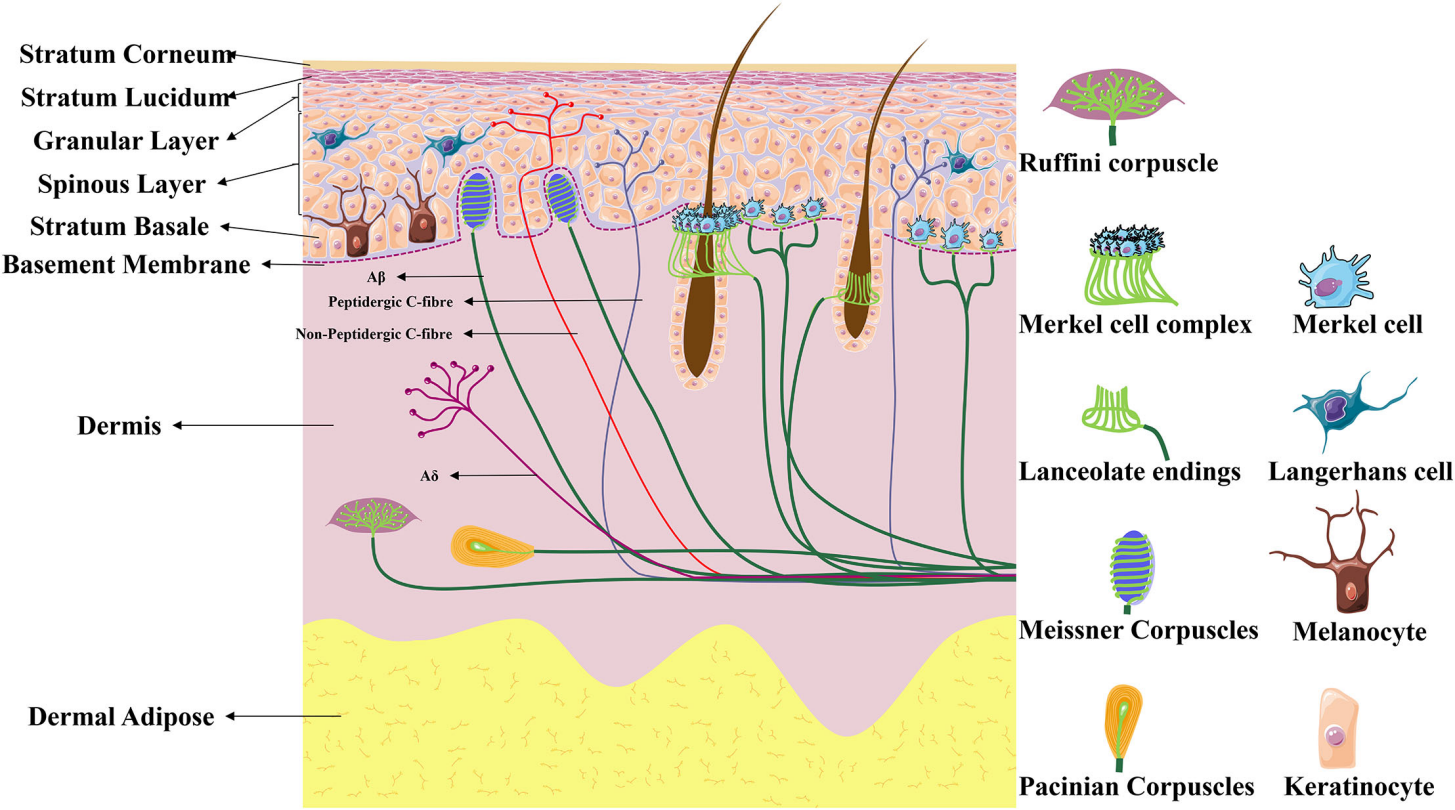
Diversity of somatosensenory neurons in the skin. Aβ-fibers in the dermis are mechanoreceptors implicated in tactile sensation. They form specialized nerve endings, as well as those innervating Merkel cells and the hair shaft. Aδ-fibers and C-fibers comprise thermoreceptors and nociceptors. Aδ-fibres terminate in the dermis, while peptidergic and non-peptidergic C-fibers terminate in different epidermal regions.

One of the main functions of skin is to perceive and respond to external stimuli and protect our body, which is mediated by the neural network on skin (21). The skin is innervated by a dense array of nerve fibers that extend throughout all its layers and associated appendages. The distribution of sensory nerves in the skin is shown in **Figure 1**. This section provides a systematic overview of the anatomical distribution and functional organization of sensory nerves within the skin.

### Nerves distribution and function

The nervous system is divided into the central nervous system and the peripheral nervous system (PNS). The PNS comprises ganglia and nerve fibers that transmit sensory information from peripheral receptors to the central nervous system, enabling the regulation of physiological functions and facilitating interaction with the external environment (24). Functionally, the PNS is categorized into somatic sensory nerves, somatic motor nerves, visceral sensory nerves, and autonomic nerves (25). The autonomic nervous system can be divided into three anatomically distinct divisions: the sympathetic nervous system, which mediates the “fight-or-flight” response; the parasympathetic nervous system, which modulates homeostatic “rest and digest” activities; and the enteric nervous system, which regulates gastrointestinal motility and secretion (21, 24, 25).

Cutaneous innervation is mainly composed of somatic sensory nerves and autonomic nerves (26). Cutaneous autonomic nerves are predominantly composed of dermal sympathetic cholinergic nerve fibers (27). These efferent fibers innervate blood vessels, lymphatic vessels, and cutaneous appendages, thereby modulating the homeostasis of cutaneous perfusion, lymphatic drainage, and appendageal activity (28, 29). However, they constitute only a minor proportion of cutaneous nerve fibers, in contrast to the dominant population of sensory nerve fibers (29).

Somatic sensory neurons derived from cranial and spinal nerves extend lengthy axons throughout the epidermal and dermal layers to detect and transmit peripheral sensory signals (6, 30). Of these, neurons with cell bodies housed within the spinal dorsal root ganglia (DRG) mediate sensory innervation to the skin of the trunk and extremities (31, 32). Additional sensory neuron cell bodies reside in the trigeminal ganglia, which provide innervation to the craniofacial skin (30–33). Cutaneous sensory nerves are classified into three main types based on myelination status and conduction velocity of action potentials: Aβ, Aδ, and C fibers. Their distinct physiological characteristics are summarized in **Table 1**. FNEs in the skin are formed by the terminal branches of myelinated Aδ fibers and unmyelinated C fibers, whereas Aβ fiber terminals associate with specialized accessory structures to form mechanosensitive tactile receptors.

**Table 1:** Main characteristics of primary sensory afferent nerves controlling human skin.

Skin position	Nerve fibers	Sensory receptor	Functions
Epidermis	C fiber Unmyelinated, 0.5–2 m/s	Thermoreceptor	Warmth
		Nociceptor	Pain
		Itches receptors	Pruritus
		Mechanicoreceptor	Stroke
Basement membrane	Aβ fiber Intermediate myelin, 35–75 m/s	Merkel cell-neurite complex	Bruising Perceiving object details Itching Pain
Dermis	Aδ fiber Thin myelin sheath, 5–30 m/s	Thermoreceptor	Cold Hot
		Nociceptor	Pain
	Aβ fiber Intermediate myelin, 35–75 m/s	Corpuscula tactus	Light touch, Vibration
		Corpuscula lamellosa	Roughness, Texture
		Ruffini corpuscles	Flexibility

### Distribution of sensory nerves in the epidermis

FNEs in the epidermis are composed of C-fibers, which extend from the subpapillary plexus, traverse the dermal papillae, and penetrate into the epidermis, forming numerous terminal branches that terminate at various epidermal levels (32, 34, 35). FNEs represent the most abundant type of sensory receptor in the skin (21). Through indirect transduction mechanisms—whereby keratinocytes or other epidermal cells detect stimuli and subsequently activate adjacent nerve terminals via paracrine signaling—these pathways mediate the perception of pain, itch, temperature changes, and certain forms of pleasant touch (36–38), as summarized in **Table 1**.

C-fibers constitute over 90% of epidermal nerve fibers and can be subdivided into peptidergic and non-peptidergic populations. Peptidergic C-fibers predominantly terminate in the spinous layer and are regulated by nerve growth factor (NGF), releasing neuropeptides such as substance P (SP) and calcitonin gene-related peptide (CGRP). Non-peptidergic C-fibers extend into the superficial granular layer, are regulated by glial cell line-derived neurotrophic factor (GDNF). These fibers also express unique markers such as the Mas-Related G protein-coupled receptor (MRGPR) and purinergic P2X receptors (39). Nociceptive neurons expressing MRGPRD exhibit close structural associations with hair follicles and epidermal keratinocytes, and their FNEs end at different depths inside the epidermis (32).

Upon entering the epidermis, a subset of Aβ fibers undergoes demyelination. Their exposed terminals are surrounded by special cells known as Merkel cells, which reside at the basement membrane. Together, these components form the “Merkel cell-neurite complex” (40). This complex functions as a slow-adapting mechanoreceptor, responsible for transmitting signals of sustained mechanical pressure to sensory nerve endings. Additionally, It is also highly sensitive to fine tactile characteristics such as an object’s shape, texture, and curvature (41). Furthermore, beyond the canonical architecture of Merkel cell-neurite complex, touch domes receive innervation from SA1 Aβ-LTMRs, Aδ-fibers, as well as peptidergic and non-peptidergic C-fibers across humans (42) and other mammals (43–45), yet their functional roles remain largely elusive.

### Distribution of sensory nerves in dermis

Myelinated sensory nerves extend from the superficial fascia to the dermal papillary layer, where they spread out into intricate nerve plexuses known as “nerve trees” (46). Aδ-fibers end in the superficial dermis, forming FNEs. Aβ nerve fibers exit the subpapillary plexus and penetrate into the deeper dermis. Within this region, they give rise to specialized tactile mechanoreceptors, including Meissner corpuscles, Pacinian corpuscles, and Ruffini corpuscles (47).

Each of these end-organs exhibits distinct sensitivity profiles toward different mechanical stimuli. Meissner corpuscles are rapidly adapting mechanoreceptors found mostly in the dermal papillary layer. They respond to low-frequency vibrations (below 50 Hz), rendering them highly sensitive to light touch and transient mechanical stimuli (48, 49). Pacinian corpuscles residing in the reticular dermis rapidly adapting mechanoreceptors. The Aβ nerve terminal is ensheathed by an onion-like lamellar capsule composed of S100-positive cells. These receptors are insensitive to sustained static pressure, yet generate transient action potentials upon abrupt mechanical deformation (40). They are extremely sensitive to high-frequency vibrations (1000 Hz), thereby underpinning the cutaneous perception of surface texture and tactile roughness (40, 49). Ruffini corpuscles located within the dermal connective tissue are slow-adapting mechanoreceptors with elongated spindle-shaped morphology. Their nerve terminals integrate with the surrounding dermal collagen matrix. Furthermore, these receptors are activated by sustained mechanical pressure and tissue compression, enabling them to demonstrate selective sensitivity to cutaneous stretch (41).

In hairy skin, the epidermal nerve plexus gives rise to a nerve network surrounding the funnel region of the hair follicle, while the dermal nerve plexus forms a circumferential innervation pattern around the isthmus portion of the hair follicle. In most mammals, including mice, hair follicles are densely innervated by lanceolate endings formed by LTMRs, which are primarily formed by Aβ, Aδ, and C fibers (50, 51). Moststudies have focused on Aβ lanceolate ends. These nerve terminals ensheath hair folliclesto form specialized lanceolate terminals, and are presumed to function as detectors of hair displacement and movement (40). In rodents such as rats and mice, Aβ slowly adapting type I (SAI)-LTMRs are only found in the face vibrissae. This enables animals to accurately perceive position, size, form, and texture of objects. However, in humans, Aβ SAI-LTMRs are connected to touch domes located between hair follicles (47, 50).

## Keratinocytes and sensory nerves

Previously, FNEs in the epidermis were believed to be the only detectors of noxious stimuli (38). However, more and more evidence (39, 52–58) has demonstrated two key findings: first, keratinocytes can modulate nociceptive signals in sensory neurons; second, they can communicate directly with these neurons via synaptic-like structures.

### Indirect regulation

**Figure 2:**
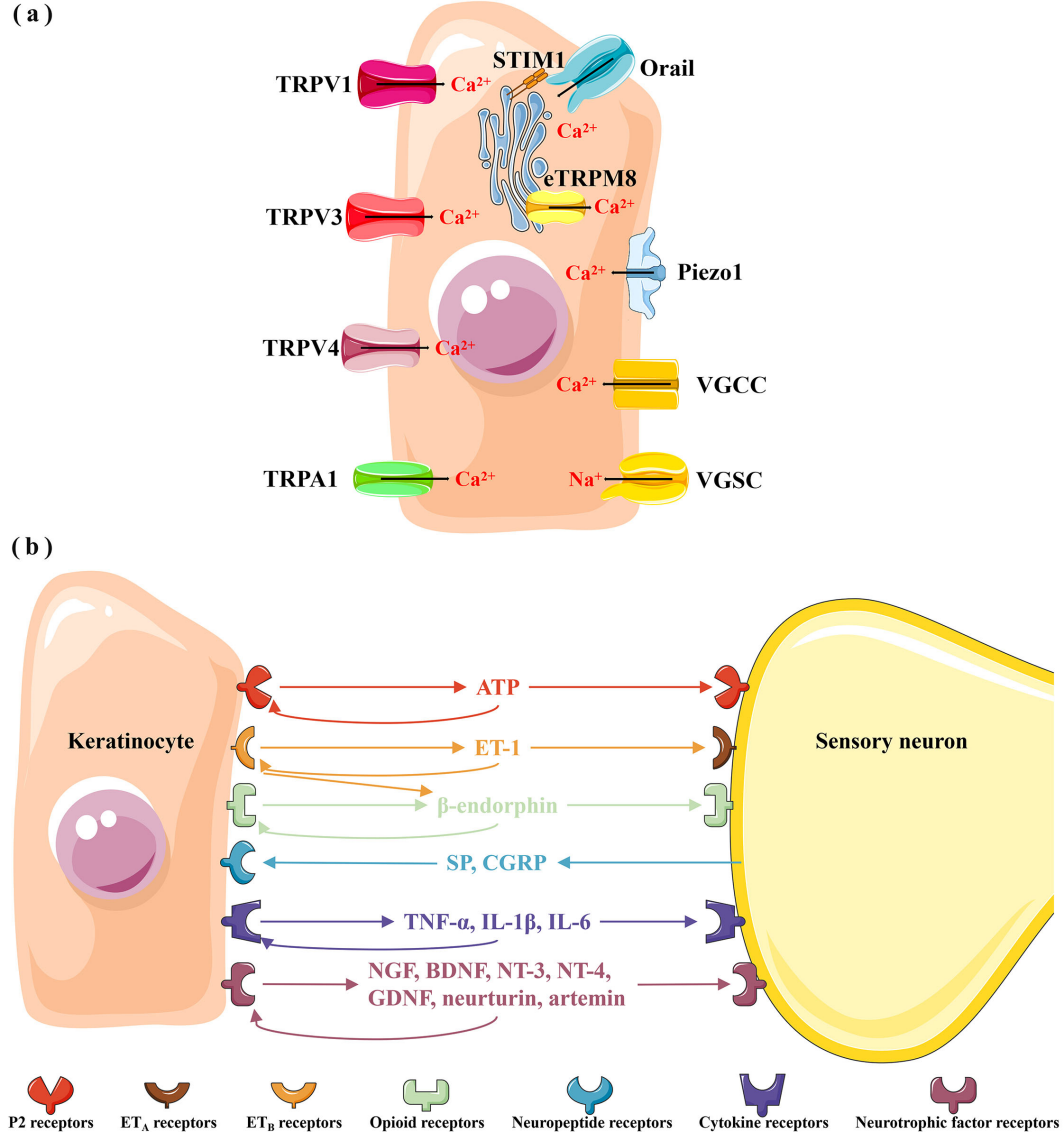
Indirect regulation between keratinocytes and free nerve endings. **(a)** External signaling molecules can activate a variety of receptors on keratinocytes, prompting them to secrete a range of chemicals. Notably, recent evidence has identified a novel mild-cold sensor protein in keratinocytes and demonstrated its localization in the membrane of the endoplasmic reticulum, which forms a Ca^2+^-permeable ion channel (66). **(b)** Keratinocytes produce neurotrophic factors (NGF, GDNF, BDNF, NT-3 and NT-4), ATP, neurotransmitters, and cytokines (IL 33, TSLP). These substances bind to specific receptors on FNEs, controlling nociceptive transmission.

Keratinocytes release many different signaling molecules, including include neurotrophic factors, adenosine triphosphate (ATP), neurotransmitters, and cytokines, which regulate nociceptive transmission via FNEs. Indirect Regulation Between keratinocytes and Sensory Neurons is summarized in **Figure 2**. Keratinocytes synthesize and secrete neurotrophic factors such as NGF, GDNF, brain-derived neurotrophic factor (BDNF), neurotrophin-3 (NT-3), and neurotrophin-4 (NT-4). Notably, NGF is secreted at the highest levels among these factors (59). Neurotrophic factors promote the survival, differentiation, and excitability of FNEs, thereby enhancing pain sensitivity (60). NGF synthesized by keratinocytes is retrogradely transported to the DRG, where it stimulates the release of neuropeptides, leading to neurogenic inflammation in the skin (61). Under conditions of stress or tissue injury, keratinocytes release cytokines such as interleukin (IL)-33 and thymic stromal lymphopoietin (TSLP), which can directly modulate sensory neurons. IL-33 activates a subset of DRG neurons by binding to its ST2 receptor, triggering Ca^2+^ influx and eliciting pruritic responses in mice (62, 63); it also directly activates FNEs to induce pain (64). TSLP activates sensory neurons expressing transient receptor potential (TRP) A1 and TSLP receptor, thereby inducing pruritus (63). Keratinocytes can release adenosine monophosphate, including antimicrobial peptides such as IL-37 and secretory leukocyte protease inhibitor, which have been shown to directly influence sensory neuron activity (8, 65).

Beyond secreting neuroactive mediators, keratinocytes express a wide range of sensory receptors (39, 53, 56, 60). Among these, TRP channels detect thermal, mechanical, and chemical stimuli. Human keratinocytes express multiple TRP channel subtypes, including TRPV1, TRPV3, TRPV4, TRPA1, and eTRPM8 (66, 67). Activation of these channels induces Ca^2+^ influx, which plays critical roles in pain perception, itch signaling, inflammation, cell proliferation, and hair follicle development (68).

The TRPV1 channel in keratinocytes not only contributes to nociceptive transduction but also participates in the pathogenesis of skin inflammation (52, 69–72). When keratinocytes are exposed to heat (70), ultraviolet (UV) radiation (71), or blue light (72), TRPV1 is activated, which subsequently promotes the release of inflammatory mediators, including cyclooxygenase-2, prostaglandin E2, IL-8, IL-1β, IL-2, IL-4, and tumor necrosis factor-α (69).

Keratinocytes utilize TRPV3 and TRPV4 for the detection of thermal heat stimuli (34, 73–78). When TRPV3 is activated, keratinocytes release ATP, which binds to the P2 receptors on sensory neurons, thereby mediating the perception of warm thermal sensations (57). TRPV3 also affects hair development in mice (79). UVB radiation activates TRPV4, thereby inducing keratinocytes produce more endothelin (ET)-1. While ET-1 can cause pain by acting on ETA receptors expressed on local nociceptors (80), it can also generate analgesia via ETB receptors. Additionally, ET-1 stimulates ETB receptors on keratinocytes, triggering the synthesis of β-endorphins, which bind to opioid receptors on nociceptors and relieve pain (81). TRPV4 can be activated by histaminergic pruritogens, which induces itch by increasing phosphorylation of mitogen-activated protein kinase (MAPK) and extracellular signal-regulated kinase in keratinocytes (ERK) (68).

TRPA1 responds to many different external stimuli. Tumor necrosis factor activates TRPA1 expression via the nuclear factor kappa B (NF-κB) and MAPK (82). which in turn increases the production of monocyte chemoattractant protein-1, IL-1α, and IL-1β, thereby amplifying inflammatory responses. However, TRPA1 expression is low in keratinocytes that are not activated. Although functional TRPM8 is expressed on the plasma membrane, an epidermal isoform of TRPM8 (eTRPM8) has been identified (66). By fine-tuning ER–mitochondrial Ca^2+^ shuttling, eTRPM8 modulates mitochondrial ATP and superoxide production to sustain epidermal homeostasis upon mild cold exposure (66). In addition, eTRPM8 regulates cold-dependent keratinocyte proliferation and differentiation (67).

Besides TRP channels, keratinocytes express other sensory receptors that participate in the modulation of cutaneous sensation, including voltage-gated sodium channels (VGSC), voltage-gated calcium channel (VGCC), adrenergic receptors, acetylcholine receptors (AChRs), and glutamate (Glu) receptors. VGSC are involved in neuropathic pain through facilitation of ATP release (83). During tissue injury or stress, catecholamines released from the sympathetic nervous system and inflammatory mediators secreted by immune cells establish a positive feedback loop via adrenergic receptor, thereby exacerbating inflammation and promoting pain sensitization (84). AChRs are classified into muscarinic AChRs and nicotinic AChRs subtypes. Keratinocytes express all five mAChR subtypes (M1–M5) as well as multiple nAChR subunits, including α3, α5, α6, α7, α9, α10, β1, β2, and β4 (56). Additionally, they express fast-acting ionotropic Glu receptors, such as N-methyl-D-aspartate, α-amino-3-hydroxy-5-methyl-4-isoxazolepropionic acid, and kainate receptors (85). VGCC (86) and Orai1/STIM1 (87–89) complexes play pivotal roles in maintaining skin homeostasis. Nevertheless, the precise mechanisms underlying their neuroregulatory functions and involvement in cutaneous sensory transduction remain largely uncharacterized and await further comprehensive exploration.

Recent studies have demonstrated that keratinocytes regulate skin mechanosensation through PIEZO1 (54). Intradermal injection of the PIEZO1 specific agonist-Yoda1 into mouse epidermis activates the PIEZO1 pathway in keratinocytes, leading to increased mechanical sensitivity of C-fibers and inducing behavioral mechanical hypersensitivity in mice (54). In contrast, a study by Rose Z. Hill et al. demonstrated that Yoda1 primarily induces acute pruritus through direct activation of somatosensory neurons, rather than indirectly via keratinocyte PIEZO1 (90). Moreover, the study revealed that PIEZO1 exerts its pruritogenic effects predominantly through the *Sst+Nppb+* subset of pruriceptive neurons, contributing to both mechanical itch and itch hypersensitivity (90). Sonoko Takahashi’s *in vivo* imaging studies demonstrated that keratinocytes and FNEs interact to induce epidermal nerve pruning, and dysregulation of this process contributes to pathological pruritus—a condition defined as chronic, intractable itching that exceeds normal physiological protective functions and is closely associated with skin inflammation, nerve damage, or systemic disorders (91).

### Direct regulation

**Figure 3:**
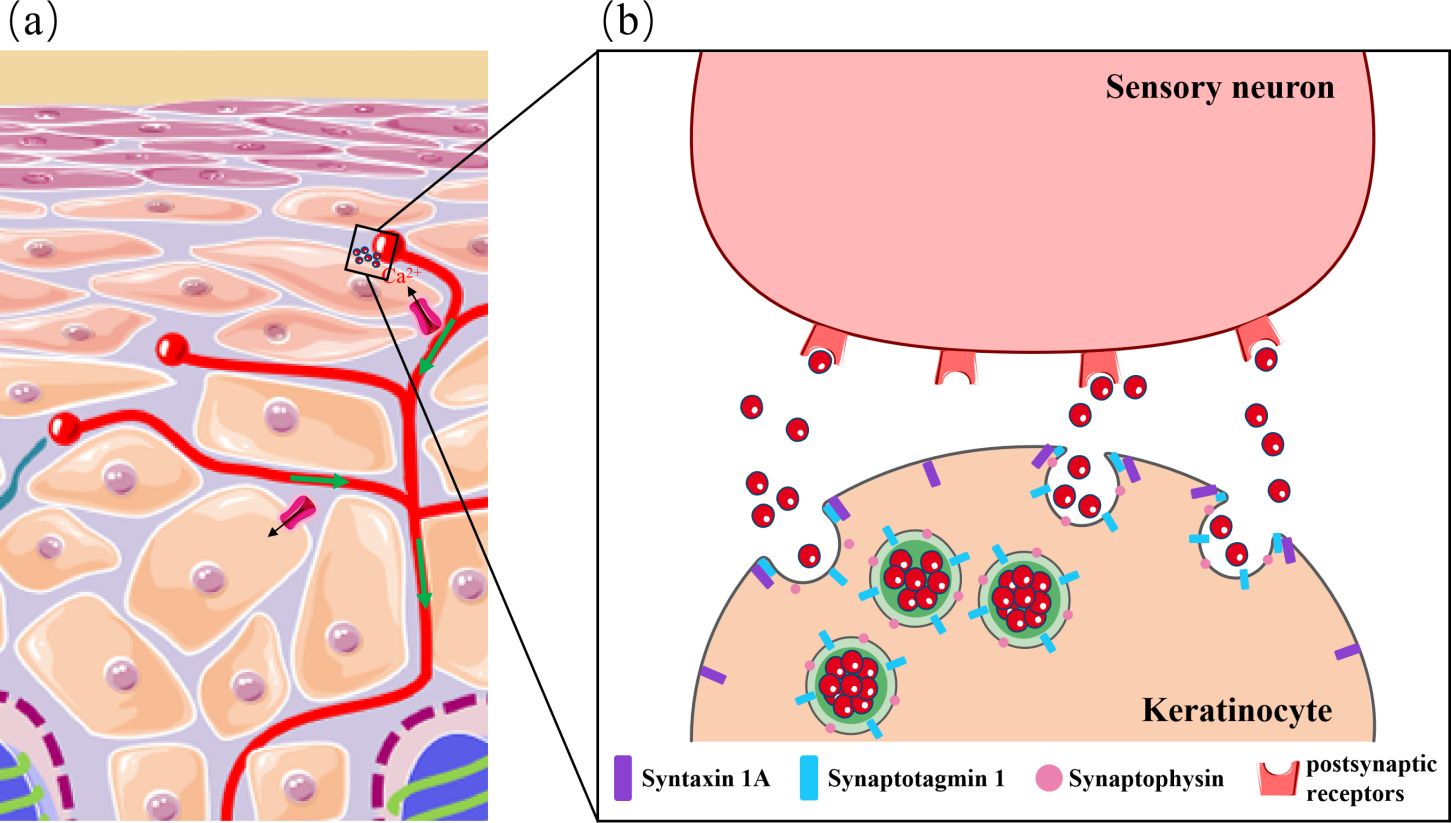
**(a)** Epidermal keratinocytes can communicate directly with sensory neurons through en passant synaptic-like contacts. **(b)** Keratinocyte TRPV1 activation elevates cytoplasmic Ca²⁺ concentration, thereby triggering vesicle exocytosis. Neuromediators released into the synaptic cleft subsequently activate sensory neurons by binding to postsynaptic receptors.

FNEs and keratinocytes have similar anatomical properties and neurotransmitter receptors (53). The above evidence support the possibility of rapid, specific paracrine crosstalk between keratinocytes and FNEs. Recent evidence indicates that keratinocytes can communicate with FNEs via synapse-like contacts, resembling the Merkel cell-neurite complex (58). This depicts the direct regulatory interaction between keratinocytes and FNEs in **Figure 3**.

Matthieu Talagas et al. (58) first reported the expression of three classic presynaptic proteins—synaptophysin, synaptotagmin-1, and syntaxin 1A—in human keratinocytes. The results from western blot and laser scanning confocal microscopy demonstrated that the key molecules essential for exocytosis—syntaxin 1A and synaptotagmin 1 (92)—are localized in transparent core vesicles as well as in components of the presynaptic active zone. Moreover, *in vitro* experiments and human skin biopsy analyses revealed pearl necklace-like clusters of presynaptic vesicle structures within the cytoplasm of keratinocytes adjacent to FNEs. Functional validation showed that when capsaicin was applied to co-cultures of human keratinocytes and rat DRG neurons, it activated the TRPV1 pathway in keratinocytes, significantly increasing neuronal currents and membrane conductance. This effect was abrogated following pretreatment of keratinocytes with botulinum toxin type C, which suppressed the capsaicin-induced enhancement in neuronal activity. These findings show that TRPV1 activation in differentiated keratinocytes of the spinous and granular layers causes vesicle exocytosis. Via this mechanism, keratinocytes release neurotransmitters that subsequently bind to postsynaptic receptors on sensory neurons.

Erbacher et al. (93) studied the ultrastructural and functional properties of the keratinocyte-nerve fiber interface in human skin. Their research revealed that epidermal nerve fiber segments are partially ensheathed by nearby keratinocytes, wherein connexin 43 may act as a molecular mediator of intercellular crosstalk. The researchers developed a co-culture setup with human sensory neurons and keratinocytes. In the human sensory neuron–keratinocyte co-culture model, neurites traverse keratinocytes intercellular grooves or are ensheathed by keratinocytes. However, no obvious SYP-positive puncta or synaptic vesicle clusters were detected in the keratinocyte cytoplasm. These results show that connexins mediate physical connection between keratinocytes and nerve fibers. Such contact constitutes a conduit for ATP-mediated signal transduction, and further modulates the transmission of sensory and pain information.

Chemogenetic and optogenetic techniques are effective tools for inducing nociceptive responses *in vivo*. By employing the *keratin 5* promoter, TRPV1 was selectively express in keratinocytes of TRPV1-knockout mice, resulting in capsaicin-specific activation of keratinocytes (52). Using this approach, they discovered that keratinocyte stimulation was sufficient to cause acute paw-licking nocifensive responses, demonstrating that keratinocyte stimulation is sufficient to evoke nociception in mice. In transgenic mice with keratinocyte-expressed halorhodopsin, yellow light promotes chloride inflow, inhibiting nociceptor action potentials and reducing behavioral responses to mechanical stimuli (55, 94). Similarly, when the light-driven outward proton pump archaerhodopsin-3 is selectively expressed in *keratin 14*-expressing keratinocytes, optogenetic inhibition of these cells attenuates reflexive behavioral responses to both cold and heat stimuli (57).

## Merkel cell and sensory nerves

**Figure 4:**
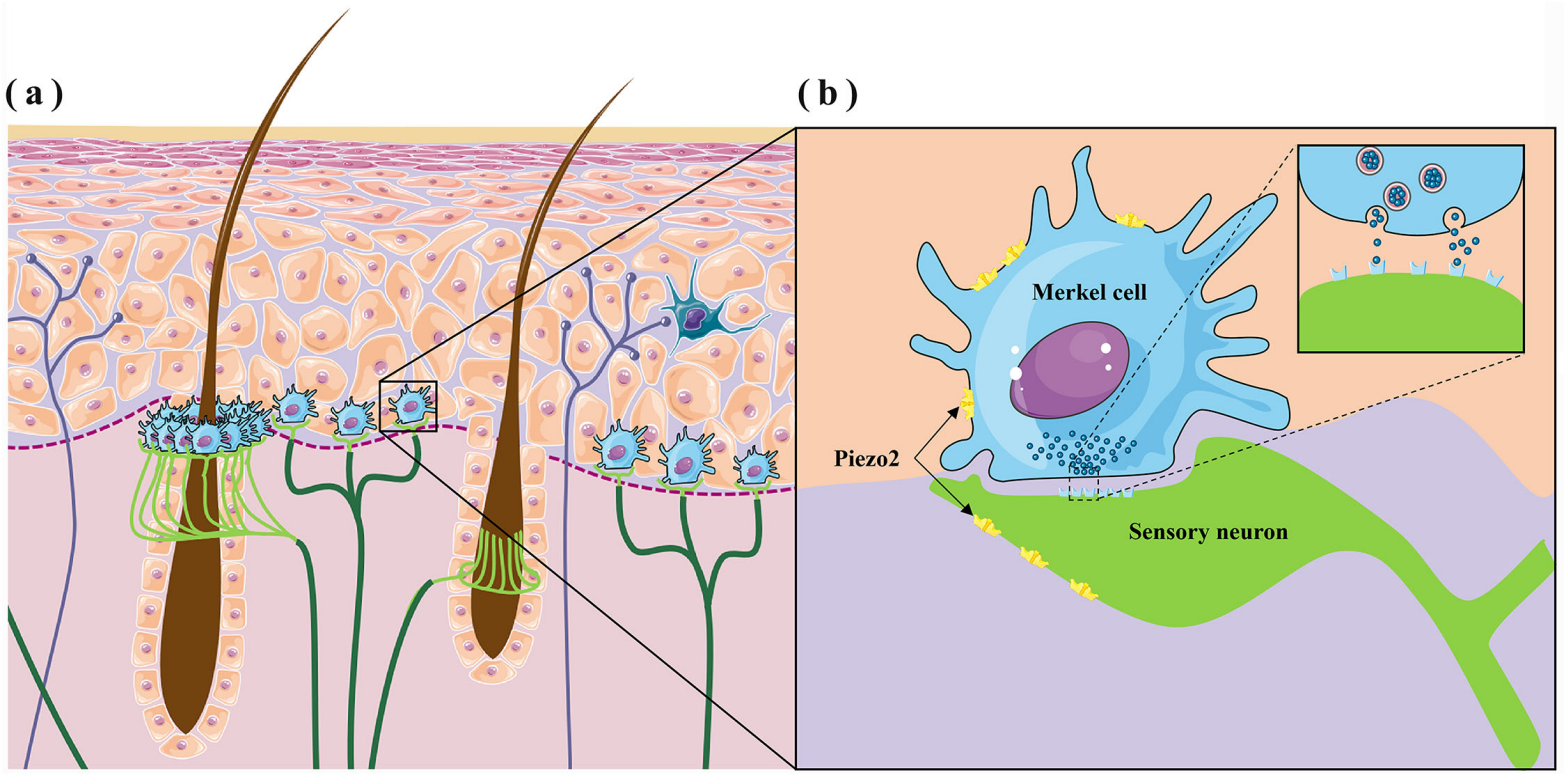
The structure of Merkel cell-neurite complex in the skin. **(a)** Merkel cells reside in the basal layer of the epidermis and are closely associated with Aβ sensory nerve. **(b)** Upon mechanical stimulation, Merkel cell releases neurotransmitters via exocytosis, activating sensory nerve terminals to trigger action potential.

In glabrous skin, Merkel cells reside in the basal layer of the epidermis, where they are anchored by desmosomes and closely associated with Aβ sensory nerve terminals (95). In hairy skin, these cells occupy specialized epidermal thickenings interspersed among hair follicles termed touch domes, where they establish synaptic junctions with sensory nerve fibers (47, 50). Merkel cells also present in hair follicle protrusions yet seldom connect adjacent nerve endings (96), and may regulate hair morphology through hair cycle-related paracrine signaling (97). Synapse-like junctions between Merkel cells and sensory afferents have been identified (98). Upon mechanical stimulation, neurotransmitters stored in dense-core vesicles are released via exocytosis, activating sensory nerve terminals to generate a SAI response (99). The structure of Merkel cell-neurite complex in the skin is shown in **Figure 4**.

### Touch

The biophysical characteristics of the touch-activated current in Merkel cells match the mechanically sensitive ion channels encoded by the *Piezos* gene (100–102). PIEZO1 and PIEZO2, the two PIEZO channel isoforms, mediate critical mechanotransduction processes (100). PIEZO2 is specifically expressed in mouse cutaneous Merkel cells but not in other epidermal cell types (101), and is also expressed in rat Merkel cells (102). Selective knockout of *Piezo2* in murine epidermal Merkel cells markedly diminishes the sustained firing rate of SAI responses mediated by the Merkel cell-neurite complex (101). These observations show that Merkel cells mechanosensitivity is entirely dependent on PIEZO2. PIEZO2 activation induces Ca^2+^ influx and neurotransmitter release from Merkel cells. This pathway triggers action potentials in Aβ afferent neurons and enhances behavioral tactile responses in rodents (102).

Merkel cells may influence SAI afferent activity by releasing excitatory neurotransmitters such as Glu, 5-hydroxytryptamine (5-HT), and norepinephrine (NE) (98). As an intrinsic receptor, the Glu receptor in Merkel cells may modulate signal transduction within these cells through a feedback mechanism (103). However, Cahusac et al. questioned whether the Glu receptor antagonists employed in this study disrupt SAI signaling via off-target inhibition of mechanosensitive ion channels, rather than through specific ligand binding to Glu receptors (104). 5-HT is thought to be a primary neurotransmitter in the Merkel cell-neurite complex. When tactile stimulation happens, rat Merkel cells in whisker follicles release 5-HT, which activates 5-HT1 receptors on SAI afferent terminals, indicating that 5-HT regulates communication between Merkel cell-neurite complexes (105). Analogous observations have further been replicated in mouse model (106). However, another study found a different outcome. Mechanical stimulation causes Ca^2+^ to influx into Merkel cells via PIEZO2 channels. It causes cells to produce NE, which binds to β2-adrenergic receptors on SAI afferents (107). This provides an alternative paradigm for neurotransmission in the complex.

Besides these classical neurotransmitters, Merkel cells also release various neuropeptides, including vasoactive intestinal peptide (VIP), CGRP, SP, methionine-enkephalin, somatostatin, and others. But these neuropeptides don’t directly affect sensory transmission. Instead, they are most likely involved in neuroendocrine or paracrine regulatory roles (108). The specific kinds of neurotransmitters and synaptic components in Merkel cells, as well as their exact functions, still need to be clarified through further research.

### Mechanical itch

Mechanical stimuli such as gentle stroking and light poking can induce cutaneous itch and trigger scratching urges. This phenomenon, termed mechanical itch, is a chronic pathological condition (109). Recent studies show that the Merkel cell-neurite complex is involved in mediating mechanical itch.

Targeted genetic ablation of Merkel cells, particularly their associated mechanosensitive Piezo2 channels mediating static Aβ-fiber activation, suffices to elicit alloknesis independent of chemical itch sensations such as histamine or chloroquine (99). Conversely, chemogenetic activation of Merkel cells enhances static firing of SA1 Aβ-LTMRs after light cutaneous mechanical stimuli and protects against alloknesis in dry skin models (110). Lu et al. explored the cellular and molecular mechanisms underlying mechanical itch caused by traditional pruritogens (111). Intradermal injection of pruritogens triggers the phospholipase C–protein kinase C-δ signaling pathway. This increases the sensitivity of the PIEZO2 channel. Finally, it promotes hypersensitivity of MRGPRA3^+^ pruriceptors to mechanical stimulation while having no effect on acute chemical pruritus. Feng et al. proved that under pathological conditions, abnormal functional connections between Merkel cells and pruriceptive C-fibers drive a self-sustaining itch-scratching cycle (112). Optogenetic activation of Merkel cells, rather than evoking SAI afferent firing mediating tactile sensation, enhanced pruriceptive C-fiber activation and spontaneous scratching in dry skin (112). Moreover, structural alterations in MRGPRA3^+^ pruriceptor terminals projecting toward Merkel cells were detected, forming a pathological touch receptor–pruriceptor circuit that drives scratch-evoked itch in mice (112).

Under physiological conditions in mice, mechanical itch circuit is gated by other Aβ-LTMRs through activation of inhibitory NPY:Cre interneurons (INs), and is independent of GRP/GRPR chemical itch pathways (113, 114). However, under pathological conditions, this gating mechanism by NPY inhibitory INs is lost, leading to alloknesis (115). Reduced serum NPY levels in pruritic psoriatic patients also support this hypothesis (116). Mechanical itch in imiquimod-induced psoriatic mice is associated with the loss of epidermal Aβ-fiber terminals, while Merkel cells remain unaffected (117). All these findings show that the Merkel cell–neurite complex plays a key role in controlling how sensitive the body is to mechanical itch.

Neurotransmitters like 5-HT and NE also influence itch perception. In pathological situations, it is important to explore whether Merkel cells mediate mechanical itch by releasing neuromodulators including 5-HT and NE. Immunohistochemical analyses has found several neurotransmitters in Merkel cells, including CGRP, SP, enkephalin A, and Glu. However, their specific roles in Merkel cell-neuron communication and itch regulation remain unclear.

### Mechanical allodynia

In cases of nerve damage or chronic inflammation, innocuous tactile stimuli can evoke pain sensations referred to as mechanical allodynia. Clinically, this phenomenon presents in three forms: dynamic mechanical allodynia elicited by light touch (e.g., fine brush), static mechanical allodynia induced by sustained pressure, and punctate mechanical allodynia triggered by localized pinprick-like stimuli using von Frey filaments. Similar to mechanical itch, mechanical allodynia is closely linked to PIEZO2. Mice that lack *Piezo2* in caudal sensory neurons fail to exhibit punctate or dynamic allodynic responses following capsaicin administration or nerve injury (118). Optogenetic activation of all *Piezo2*-expressing neurons in mice induces nocifensive behaviors such as paw withdrawal, defensive movements, and licking (118). The Merkel cell-neurite complex contributes to mechanical allodynia after peripheral nerve injury in a sex-dependent manner, with such pain responses observed exclusively in male mice (119). PIEZO2 is expressed in both neuronal and non-neuronal cells within the PNS of rats. Inflammatory conditions and nerve injury enhance PIEZO2-mediated mechanical sensitivity, suggesting that PIEZO2 not only functions in the detection of external and internal mechanical cues but may also participate in additional intercellular or intracellular signaling pathways within pain circuits (120). Xie et al. discovered that endorphin A2 interacts with kinesin family member 5B, a molecular mechanism underlying mechanical hyperalgesia. This interaction mediate membrane trafficking of PIEZO2 in DRG neurons, thereby affecting tactile and mechanical allodynia (121). In the PNS, pro-inflammatory mediators such as bradykinin and the second messenger Epac1 enhance PIEZO2 channel function downstream, potentiating the sensitivity of sensory neurons (122, 123).

Szczot et al. conducted quantitative sensory tests on human patients with *Piezo2* loss-of-function mutations. When local inflammation was produced, these patients shown no mechanical hypersensitivity to gentle stimuli such as a fine brush, air puff, or vibration (124). Findings from human studies and animal models complement one another, collectively demonstrating that neuronal PIEZO2 is necessary for the development of mechanical allodynia.

## LCs and FNEs

**Figure 5:**
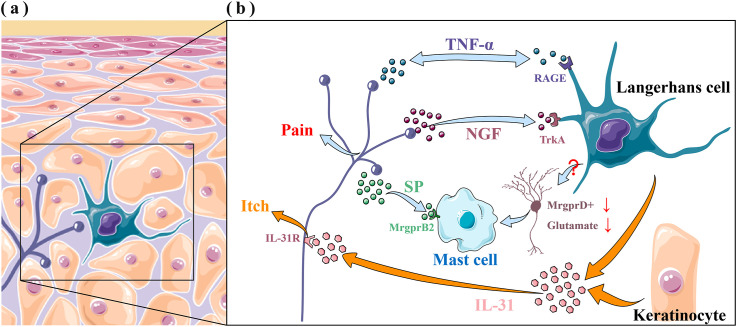
The interplay between langerhans cells and free nerve endings. **(a)** Langerhans cells in the epidermis can form close associations with free nerve endings. **(b)** Langerhans cells and free nerve endings engage in bidirectional communication through signaling molecules such as neuropeptides, neurotrophic factors, and cytokines.

LCs are immunological cells found in the epidermal spinous layer with a distinctive star-like appearance (125). LCs dynamically extend and retract dendrites between keratinocytes (126), enabling them to survey antigens across multiple epidermal layers without compromising skin barrier integrity (127). Furthermore, LCs can form close associations with peripheral sensory nerve endings (**Figure 5a**).

### Functional differentiation of LCs

LCs have obvious similarities with dendritic cells and macrophages (125, 128). Like tissue-resident macrophages, LCs in the epidermis are maintained by self-renewal through exhibiting a slow proliferation rate to replace dying and emigrating cells under homeostatic conditions. However, unlike most macrophages, LCs possess dendritic cell-like migratory capacity (128). During both steady-state conditions and inflammation, LCs can migrate across the epidermal basal layer to reach lymphatic vessels and subsequently travel to regional lymph nodes, where they present antigens to cells of the adaptive immune system. This migratory process is initiated by the downregulation of E-cadherin, enabling LCs to detach from neighboring keratinocytes (129). Upon reaching the basement membrane, LCs locally secrete collagen-degrading matrix metalloproteinases 2 and 9, facilitating dermal remodeling and enabling transdermal migration of LCs (130). Once within the dermis, LCs can enter the lymphatics in order to migrate to regional lymph nodes, to initiate adaptive immune responses through antigen presentation.

Seré et al. identified two distinct subsets of LCs: long-term LCs, derived from myeloid precursors during steady-state conditions, and short-term LCs, arising from Gr-1^hi^ monocytes during inflammation (131). The development of long-term LCs is dependent on the DNA-binding inhibitor 2, whereas short-term LCs do not require binding inhibitor 2 expression (131). Following UV irradiation, short-term LCs are initially recruited to the epidermis and are gradually replaced by long-term LCs, thereafter reside stably in the epidermis (131). Short-term and long-term LCs show distinct phenotypes and morphologies. Langerin, EpCAM, CD24, and CD205 are among the LC markers with distinct expression patterns, all of which are highly expressed in long-term LCs. However, short-term LCs express both CD11b and CX3CR1 (132). Skin immune homeostasis relies heavily on LCs, which engage in active crosstalk with other skin cells, including keratinocytes and T lymphocytes, thereby facilitating wound healing (127, 133–140).

### Communication between LCs and FNEs

FNEs modulate LC function by releasing neuropeptides such as VIP and pituitary adenylate cyclase-activating polypeptide. These neuropeptides directly interact with LCs and attenuate their antigen−presenting capacity within draining lymph nodes (141). Loss of epidermal innervation impairs LCs activation and inhibits their migration, leading to an accumulation of LCs in the epidermis (142). Similarly, spinal cord injury patients have permanently denervated skin, which compromises epidermal immune protection and results in aberrant accumulation of LCs (143). However, functional electrical stimulation was insufficient to reverse epidermal LC density and restore skin immunocompetence (143).

As shown in **Figure 5b**, Langerhans cells and free nerve endings engage in bidirectional communication via signaling molecules including neuropeptides, neurotrophic factors, and cytokines, which is critical for preserving cutaneous innervation and normal sensory function. Depletion of LCs leads to a reduction in the number of skin nerves; specifically, it results in the loss of MRGPRD-expressing FNEs, thereby causing mast cell hypersensitivity and skin inflammation (144, 147). LCs express cannabinoid receptor 2, and administration of cannabinoid receptor 2 receptor agonists has been shown to alleviate mechanical allodynia in models of chemotherapy-induced peripheral neuropathy (145). Pain is a common complication of diabetes. NGF exerts its effects via tropomyosin receptorkinase A (Trk-A) receptors expressed on LCs; these receptors interact with neurons via the receptor for advanced glycation end-products (RAGE), thereby contributing to mechanical allodynia in a mouse model of type 2 diabetes (146). LCs play an important part in causing itch by producing IL-31 (181).

## Melanocytes and sensory nerves

Melanoblasts, which originate in neural crest cells, migrate into the epidermis and hair follicles during skin development. There, melanocytes turn into mature melanocytes. Possessing a dendritic morphology, mature melanocytes synthesize melanin within melanosomes, transfer pigment to adjacent keratinocytes, and then undergo apoptosis (148). Melanocytes and skin sensory neurons share a common embryonic origin: the neural crest. Both cell types are regulated by comparable signaling pathways, including ET and neurotrophic factor signaling, and utilize similar molecular cascades, such as protein kinase C and pro-opiomelanocortin-p53-dependent pathways (149). Furthermore, melanocytes and sensory neurons have direct physical contact owing to their close anatomical proximity (150). Collectively, these inherent shared developmental, molecular, and anatomical similarities underpin crucial functional crosstalk between the two cell types. These shared characteristics imply two key implications: first, skin sensory neurons can modulate melanocyte activity, which in turn regulates both skin and hair pigmentation; second, melanocytes could be employed as a biological model to investigate neurodegenerative processes.

Melanocytes make direct contact with FNEs in human senile lentigines. At the same time, sensory neurons show significant upregulation of repulsive guidance molecule B expression (150). Co-culture experiments show that sensory neurons secrete the repulsive guidance molecule B protein, which promotes melanocyte survival and melanin formation (150). Via this approach, sensory neurons actively govern skin pigmentation. Neurons also influence hair color by controlling melanocyte stem cells (McSCs). Upon stress-induced activation of the sympathetic nervous system, NE is released, which drives rapid proliferation, differentiation, and migration of McSCs. This process accelerates premature McSCs exhaustion, ultimately leading to hair graying (151).

## Conclusions

The skin is the largest organ in the human body. Beyond serving as a barrier against external insults, it dynamically maintains systemic homeostatic balance. Here, cutaneous nerves play a crucial role in maintaining skin integrity and regulating systemic responses to diverse pathological conditions. Epidermal cells and sensory nerves share analogous developmental origins and anatomical proximity, which provides a structural foundation for intercellular communication. Sensory neurons and epidermal cells can communicate indirectly through soluble mediators, as epidermal cells express neural receptors and release a variety of neuroactive substances. Furthermore, some epidermal cells form synaptic connections with sensory nerve terminals, thereby facilitating direct communication between nerves and epidermal cells. This review describes the distribution and functional features of cutaneous sensory nerves, as well as their bidirectional crosstalk with epidermal cells and nerve cells. Key regulatory processes include mutual regulation of keratinocytes, Merkel cells, LCs, melanocytes, and sensory neurons.

Interactions between neuro-epidermal cells are closely associated with many skin diseases, including psoriasis (152–155), atopic dermatitis (156–163), and neurodermatitis. Additionally, emerging evidence shows that skin disorders may increase the risk of developing neurodegenerative diseases, such as Alzheimer’s (164–172) and Parkinson’s (173–180) disease. Current research confirms a bidirectional relationship between skin disorders and neurological conditions; however, the underlying pathophysiological mechanisms remain poorly understood. More studies are needed to make these complex molecular and cellular pathways clear. So future research should keep focusing on two main areas. First, consider how epidermal nerves accurately govern immune surveillance, inflammation management, and anti-aging mechanisms. Second, examine how these regulatory relationships become dysregulated in disease settings, contributing to the start and progression of complex pathological diseases.

High-throughput technologies, including as genomics, proteomics, and single-cell sequencing, have opened up previously unexplored avenues for understanding neurocutaneous connections. These approaches enable researchers to precisely map the neural-epidermal crosstalk across immune regulation, inflammatory responses, and cutaneous aging processes. These technologies also lay the foundation for early diagnosis, personalized treatments, and novel drug development. Moving forward, interdisciplinary cooperation and open data sharing will be indispensable for resolving this scientific challenge. Integrating multidisciplinary expertise will facilitate comprehensive elucidation of fundamental physiological and pathological mechanisms in humans.
